# Acute Hypertensive Heart Failure Due to Post-anesthetic Shivering After Mitral Valve Transcatheter Edge-to-Edge Repair: A Case Report

**DOI:** 10.7759/cureus.82518

**Published:** 2025-04-18

**Authors:** Shintaro Suzuki, Hiromitsu Kuroda, Nobutaka Nagano, Ryo Nishikawa, Mitsutaka Edanaga, Hiroomi Tatsumi, Satoshi Kazuma

**Affiliations:** 1 Department of Intensive Care Medicine, Sapporo Medical University, School of Medicine, Sapporo, JPN; 2 Department of Cardiovascular, Renal and Metabolic Medicine, Sapporo Medical University, School of Medicine, Sapporo, JPN; 3 Department of Anaesthesiology, Sapporo Medical University, School of Medicine, Sapporo, JPN

**Keywords:** afterload mismatch, hypertensive heart failure, mitraclip®, post-anesthetic shivering, transcatheter edge-to-edge repair

## Abstract

Post-anesthetic shivering (PAS) is common after general anesthesia and causes sympathetic excitement, followed by elevated blood pressure. Mitral valve transcatheter edge-to-edge repair (TEER) with MitraClip® (Abbott, Santa Clara, CA) increases left ventricular output resistance because of mitral valve narrowing. PAS after TEER synergistically raises cardiac load, surpassing the left ventricular working reserve and greatly increasing the risk of left ventricular failure in patients.

A 64-year-old woman diagnosed with functional severe mitral regurgitation underwent implantation of MitraClip under general anesthesia and was subsequently transferred to the intensive care unit (ICU). Thirty minutes after admission to the ICU, the patient exhibited shivering, elevated blood pressure, and reduced oxygen saturation. There was no evidence of clip displacement, and the cause of this oxygenation impairment was considered to be hypertensive heart failure triggered by shivering. Noninvasive mechanical ventilation, antihypertensive medication, and body surface warming were initiated. The patient showed signs of recovery within two hours.

Shivering can increase patient risk after TEER. Effective prevention of shivering is essential because TEER, along with shivering, can increase left ventricular ejection resistance, known as afterload mismatch.

## Introduction

Mitral regurgitation (MR) is the most prevalent valvular heart disease, particularly in older patients [1]. Patients requiring mitral valve surgery often have multiple comorbidities and are high-risk surgical cases [1]. Particularly, in functional mitral regurgitation (FMR) caused by left ventricular (LV) dysfunction, there is a reciprocal relationship between FMR and LV dysfunction, in which LV dilation and reduced contractility exacerbate FMR. Therefore, a fundamental approach must be developed to manage FMR refractory to guideline-directed medical therapy to prevent further LV overstretch [2].

Thus, transcatheter edge-to-edge repair (TEER) using MitraClip® (Abbott, Santa Clara, CA) is a significant advancement in cardiology, specifically for transcatheter mitral valve (MV) repair in patients with high-risk MR. MitraClip is the first interventional device for TEER, which was approved by the CE Mark in 2008 and by the U.S. Food and Drug Administration in 2013, and its use has steadily increased [3], making it a key option for patients unsuitable for surgery. TEER with Mitra Clip was authorized for marketing in Japan by the Pharmaceuticals and Medical Devices Agency on October 31, 2017 [4].

TEER has gained recognition as a percutaneous treatment option for patients with symptomatic severe MR who have an elevated risk with conventional surgical intervention [5]. The procedure is often implemented with radiographic fluoroscopy and transesophageal echocardiography and is performed mainly under general anesthesia [6]. Anesthesiologists are increasingly confronted with TEER, which positions them to play a vital role in the care of high-risk patients.

Here, we report a case of acute hypertensive heart failure triggered by post-anesthetic shivering (PAS) after TEER with MitraClip. PAS is a common complication that can occur after general anesthesia, particularly when remifentanil is administered [7]. The underlying mechanism of its occurrence is not exclusively thermogenic; it can also be non-thermogenic, resulting from pain or surgical invasion [7]. PAS increases the patient’s discomfort and surgical pain due to muscle rigidity, oxygen consumption, and catecholamine release, which increase heart rate, cardiac output, vasoconstriction, and blood pressure [7]. In addition, most patients requiring mitral valve TEER have stretched myocardium and a subsequently dilated MV due to diminished LV contractility. Therefore, PAS can cause decompensated heart failure in patients with compromised cardiopulmonary reserves [8]. However, additional factors specific to TEER should be considered.

## Case presentation

A 64-year-old woman, 160.5 cm tall and weighing 72.4 kg, with a BMI of 28.1 kg/m^2^, presented with a complex medical history. Her condition had deteriorated due to diastolic phase hypertrophic cardiomyopathy; as a result, she was diagnosed with non-ischemic dilated cardiomyopathy and managed under guideline-directed medical therapy, including an implantable cardioverter defibrillator. She also employed a home nasal continuous positive airway pressure device to manage her sleep apnea syndrome. She was admitted to our hospital because of a heart failure recurrence. Transthoracic echocardiography revealed severe functional MR, with reduced left ventricular ejection fraction (LVEF) of 34%. Considering her low LVEF and elevated surgical risk, she was scheduled for TEER with MitraClip implantation under general anesthesia.

**Figure 1:**
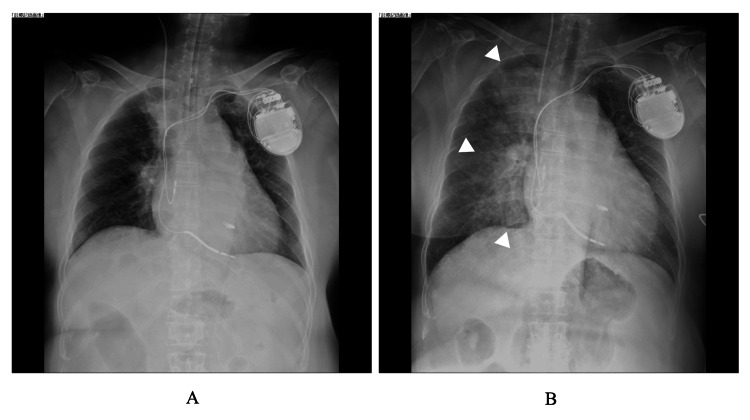
Chest radiograph at the end of transcatheter edge-to-edge repair with MitraClip® procedure (A), at the onset of hypertension and shivering (B). The butterfly shadow (△) is more pronounced at the symptom onset than at the end of the procedure.

The patient was induced with 3 mg IV midazolam for sedation, 0.16 μg/kg/min remifentanil for analgesia to minimize the hemodynamic instability, and 50 mg rocuronium for muscle relaxation and then underwent tracheal intubation. Anesthesia was maintained using sevoflurane 1% and remifentanil 0.05 µg/kg/min. During anesthesia, dobutamine was administered at 4.5 µg/kg/min with continuous intravenous infusion to stabilize hemodynamics. For hypotension, phenylephrine was administered intravenously in 0.1 mg doses as needed to maintain the patient's blood pressure. The TEER procedure was successfully performed, reducing significant MR to trivial. Intraoperative hemodynamics were generally stable, and 100 µg of fentanyl was administered just before the end of surgery for postoperative pain management. Body temperature was 36.5°C at induction of anesthesia and 36.0°C at discharge. Postoperative chest radiography revealed no significant congestion (Figure 1A). Consequently, continuous remifentanil infusion was discontinued, and a muscle-relaxant antagonism protocol involving sugammadex was initiated. The patient was successfully weaned off the ventilator, and extubation was performed smoothly. Intraoperatively, the patient received 2250 mL of fluids (1700 mL Ringer's acetate, 270 mL saline, and 280 mL packed red blood cells). Intraoperative blood loss was 50 mL, and urine output was 1300 mL. Therefore, the intraoperative total fluid balance was + 900 mL. The total operative time was 170 min, and the duration of anesthesia was 271 min. The patient was transferred to the intensive care unit (ICU).

**Figure 2:**
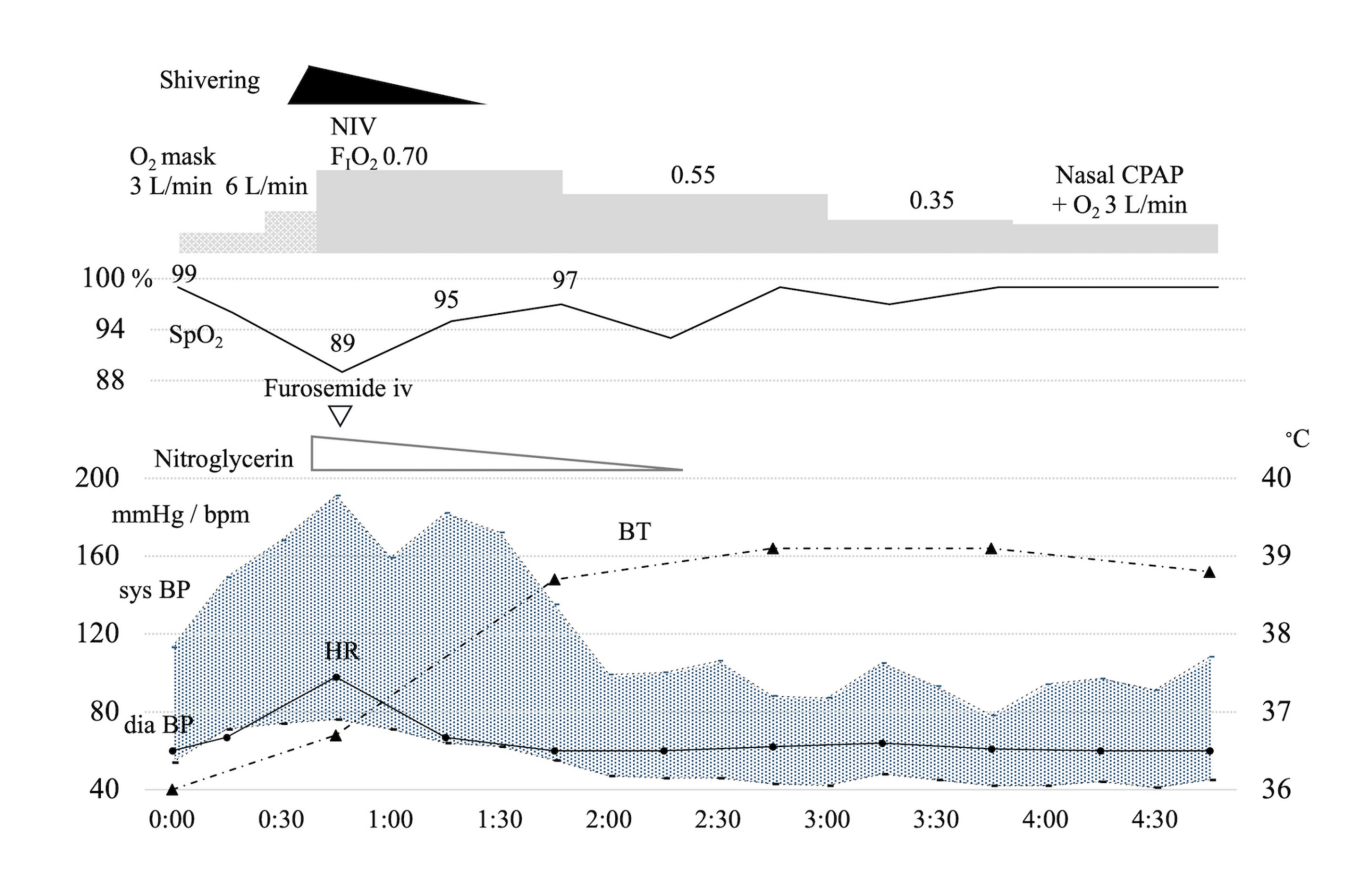
Time course of treatment of acute hypertensive heart failure after ICU admission. F_I_O_2_, fraction of inspiratory oxygen concentration; SpO_2_, oxygen saturation of peripheral artery; sys BP, systolic blood pressure; dia BP, diastolic blood pressure; CPAP, continuous positive airway pressure; HR, heart rate; BT, body temperature The patient exhibited a decrease in SpO_2_ accompanied by an increase in blood pressure and the onset of shivering. In response, noninvasive ventilation (NIV) was instituted to offer respiratory support, and nitroglycerin and furosemide were administered. Furthermore, the body warming apparatus was activated. After two hours, shivering subsided, blood pressure normalized, and oxygenation levels improved.

Approximately 30 min after admission to the ICU and 45 min after remifentanil administration was discontinued, the patient suddenly exhibited noticeable shivering, followed by an elevation in blood pressure to 168 / 74 mmHg, and an impaired oxygenated peripheral oxygen saturation of 92% (Figure 2). Chest radiography showed notable findings of pulmonary edema (Figure 1B). At that time, she had no complaints of pain. Bedside transthoracic echocardiography revealed no evidence of clip displacement, and MR was trivial, leading to a diagnosis of hypertensive heart failure; the post-TEER LVEF was also 35%, which was identical to the value of pre-TEER. TEER clipping created two MV orifices. Before-and-after transesophageal echocardiography tests demonstrated that the valve area changed from 4.70 cm^2^ to 1.26 cm^2^ and 1.30 cm^2^ for the two orifice areas, and the mitral pressure gradient increased from 0.8 mmHg to 2 mmHg for both. The noninvasive positive pressure ventilation was maintained with a high fraction of inspiratory oxygen of 70%, and intravenous nitroglycerin and furosemide were administered subsequently. Furthermore, body surface heating was conducted using a warm-air heating device to mitigate the incidence of shivering.

One hour after the onset of shivering, the patient's body temperature rose to 38.5 °C, blood pressure returned to normal of 135 / 55 mmHg, and oxygenation showed significant improvement. Three hours after the onset of shivering, adequate oxygenation was achieved. At that point, continuous nasal positive airway pressure (CPAP) was initiated with supplemental oxygen for the initial episodes of sleep apnea syndrome. The patient was transferred from the ICU to the general ward the following day.

## Discussion

In this report, we discuss a case of hypertensive heart failure accompanied by postoperative cardiogenic pulmonary edema following PAS under general anesthesia during TEER. Remifentanil-induced PAS significantly worsens cardiogenic pulmonary edema. Remifentanil is well known to cause shivering, with a relative risk of approximately double that of fentanyl [8, 9]. This side effect is more frequent at higher doses but has also been observed at lower doses [8, 9]. PAS associated with remifentanil involves two mechanisms. The first is thermoregulatory shivering, in which opioids increase the threshold for shivering [10]. The second is non-thermoregulatory shivering, triggered by opioid withdrawal because of a rapid decrease in blood remifentanil levels, resulting in increased pain stress from the loss of μ-receptor stimulation [10]. The non-thermoregulatory shivering may occur even after cessation of low-dose remifentanil administration [11]

In the present case, the patient's body temperature upon ICU admission was 36°C, which could be semihypothermia, because the clinical practice guidelines proposed by the National Institute for Health and Care Excellence recommend that the postoperative patients’ core body temperature should be preserved at > 36.0°C in the recovery room [12]. In general, the core temperature setpoint is raised by surgical incision [13]. Furthermore, remifentanil elevates the shivering threshold, potentially triggering PAS. Additionally, various factors can be involved in postoperative cardiogenic pulmonary edema, especially the TEER for FMR. 

The transesophageal echocardiographic findings after TEER indicated that MR flow and the valve area were reduced, confirming the expected therapeutic effect of TEER on MR. Potential factors involved in cardiogenic pulmonary edema after TEER for FMR must be identified. The following three key considerations were considered: (1) afterload mismatch, (2) excessive preload, and (3) thermal control issues inducing PAS during TEER. 

First, acute LV dysfunction following surgical MR treatment is widely recognized as an afterload mismatch [14]. MVR can increase the LV afterload and impair systolic function by obstructing low-resistance regurgitation in the left atrium. Patients with FMR demonstrate reduced LV contractility; similarly, TEER in FMR may lead to afterload mismatches. A retrospective study of 73 patients with FMR treated with TEER revealed that afterload mismatch, indicated by a sudden drop in the LV ejection fraction, occurred in 19 patients shortly after the procedure [15]. Those with afterload mismatches had significantly larger end-diastolic and end-systolic diameters than those without afterload mismatches. Therefore, careful management of the preload and afterload following TEER remains crucial.

Second, the volume of irrigation fluid used during intravascular catheter manipulation is uncertain, and ventilatory weaning significantly affects the preload. Irrigation fluid introduced at the catheter insertion site helps prevent air embolism. However, it is difficult to determine the injected intravascular volume accurately. Onimaru et al. reported on a difficult case capturing the valve leaflets during TEER with MitraClip® procedure, suggesting that the intravascular irrigation fluid dose, which is difficult to quantify, may have been excessive [16]. 

Additionally, weaning from mechanical ventilation to spontaneous breathing may trigger weaning-induced pulmonary edema [17]. Spontaneous breathing alters the intrapleural pressure from positive to physiological negative values, enhancing an increase in venous return and preload. The patient developed diastolic dysfunction because of diastolic phase hypertrophic cardiomyopathy, suggesting that her cardiac function could have been susceptible to increased preload. Hypoxemia is the most common postoperative complication in patients with diastolic dysfunction due to a fluid shift to the central blood volume after general anesthesia [18]. The interplay of these preload factors may have resulted in a synergistic effect, leading to an augmentation in both the preload and afterload owing to shivering.

Third, the inherent characteristics of TEER with the MitraClip procedure present challenges in maintaining adequate temperature control during anesthesia. A major issue is that X-ray fluoroscopy guidance is necessary for catheter manipulation, which has limitations in areas where body surface-warming devices can be applied. Additionally, in endovascular interventions performed under X-ray fluoroscopic guidance, the hybrid operating room (OR) temperature is often kept lower than in standard surgical procedures, typically maintained at 21-23°C. According to a report by Lee and Lee, the hybrid OR has a higher incidence of postoperative hypothermia than the standard ORs [19]. These factors facilitate shivering after remifentanil administration.

Recognition of the abovementioned issues is essential for performing TEER under general anesthesia, especially in patients with FMR caused by low LV function. Careful attention should be paid to avoid abrupt changes in preload and afterload, as low LV function persists even after clip implantation. Considering these risks can significantly improve patient outcomes and safety.

The following are potential prophylactic methods for preventing PAS following TEER used in the treatment of FMR. Since PAS can occur a few hours after discontinuing remifentanil administration, we recommend the implementation of the following strategies: (1) continue mechanical ventilation and sedation for a few hours to ensure adequate monitoring for afterload mismatch caused by TEER, as well as increased preload and afterload associated with PAS; (2) maintain adequate body surface warming; and (3) administer meperidine, which is recommended to prevent PAS induced by remifentanil. These methods aim to manage complications and enhance patient comfort during recovery effectively.

## Conclusions

In conclusion, we encountered a patient who underwent TEER with MitraClip implantation who developed acute hypertensive heart failure triggered by PAS. Sympathetic excitation in the postoperative period, such as PAS, can increase systemic vascular resistance and cause peripheral vasoconstriction. This can lead to an increase in venous return, ultimately resulting in postoperative heart failure. In addition, TEER itself can cause significant cardiovascular changes in the preload and afterload. A patient treated with TEER for FMR may be sensitive to hemodynamic changes. Sympathetic excitation during the postoperative period can increase systemic vascular resistance, resulting in postoperative heart failure. Therefore, anesthesiologists should be concerned about the inherent risks associated with TEER and PAS.
